# Tetanus

**Published:** 1902-05

**Authors:** John J. Repp

**Affiliations:** Ames, Iowa


### REPORTS OF CASES.

### TETANUS.

By John J. Repp, V.M.D.
AMES, IOWA.

Case I.—Bay gelding, ten years old, 16 hands high, 1300 pounds weight, used for draying.

History. On Saturday, January 18, 1902, while at work the horse tread upon a tenpenny nail, which penetrated the left posterior foot about the middle of the lateral lacuna. The animal did not show lameness visible to the owner until the next morning, when the nail, which remained in the foot, was seen and removed. It had penetrated about an inch and then bent over, the upper portion of the nail lying in the lacuna. The animal was kept at his usual work until Thursday evening, January 23. On the evening of this day he ate his feed as usual. As a result of inquiry the owner could recall that on Thursday the horse did not work in his usual manner. Although high-spirited, he lagged a little, and his ears were not moved as much as usual. It is inferred from this that the horse was showing slight symptoms of tetanus at this time. On the morning of Friday, January 24, the horse did not eat, was excitable, held his head stiffly, and his jaws were set. This led the owner to suspect tetanus.

Personal Observations. I saw the animal first at 1 p.m., Friday, January 24. His jaws were firmly set; ropy saliva hanging from mouth; head extended; muscles of neck prominently defined; membrana nictitans slightly protruded; gait stiff; temperature, respiration, and pulse normal.

Treatment. The stabling was first class, so needed no attention. Feeding of gruel by the mouth was attempted by means of a funnel and hose, but had to be given up because the horse could not swallow. The medication consisted of one ounce of chloral dissolved in half-pint of acacia solution, per rectum, and five grains of morphine sulphate, subcutaneously three times daily. The nail wound was enlarged and pure creolin applied to it. Although the drugs brought about a slight temporary amelioration of the symptoms, the animal grew gradually worse until it died, on the night of Saturday, January 25. This case is interesting, as will be noticed, chiefly because of the short incubation period—five days—and the short course—about two days. The great power and rapid progress of the morbid process were very obvious.

CASE II.—Bay gelding, three years old, grade with some standard blood.

HISTORY. This colt had been castrated by a farmer about May 10, 1901. About June 1 the owner noticed some signs of illness, and finally (June 10) brought the animal to the Veterinary Hospital, Iowa State College, where it came under my care.

I examined the colt immediately after its arrival from a twelve-mile journey on foot. The colt was wild, as it had never been handled, and was very excitable. On approaching its head it would wheel around and try to get away. It was very stiff, head extended, tail raised, and the membrana nictitans drawn the greater part of the time almost completely over the eye. The owner said there was "something wrong with the horse's eyes" which he wished attended to. The castration wounds had healed. A diagnosis of tetanus was made and the owner given an unfavorable prognosis. He consented to leave it in the hospital for treatment.

TREATMENT. The colt could chew and swallow, so it got all it needed to eat, although it ate with much difficulty. The medicinal treatment instituted consisted of five grains of morphine sulphate twice a day subcutaneously and one ounce of chloral in acacia solution by the mouth. The drenching produced such great excitement, at one time causing the horse to fall to the floor, that it was abandoned after two or three attempts, and the chloral given per rectum. After about one week of treatment the chloral was discontinued, but the morphine injections were kept up until June 28. On this date the colt was considerably improved, though it still showed marked evidence of the disease. The owner now wished to remove the animal from the hospital to a nearby farm in order to diminish cost of keeping. This was done and medication discontinued. After a week's stay at this farm the colt was taken home almost entirely well. A letter from the owner, dated March 15, 1902, states that the animal made a complete recovery, and has since remained in good health. The marked features of this case seem to be chronicity of the attack, which was quite virulent in all its features except the lack of severe involvement of the organs of mastication and deglutition, and the large quantity of morphine which the animal received.

---

Dr. J. G. Bethune, of Punxsutawney, Pa., has one of the largest veterinary hospitals in Western Pennsylvania.

